# Structural Variations of Human Glucokinase Glu256Lys in MODY2 Condition Using Molecular Dynamics Study

**DOI:** 10.1155/2013/264793

**Published:** 2013-02-13

**Authors:** Nanda Kumar Yellapu, Kalpana Kandlapalli, Koteswara Rao Valasani, P. V. G. K. Sarma, Bhaskar Matcha

**Affiliations:** ^1^Division of Animal Biotechnology, Department of Zoology, Sri Venkateswara University, Tirupati, Andhra Pradesh 517502, India; ^2^Department of Biochemistry, Sri Venkateswara Institute of Medical Sciences, Tirupati, Andhra Pradesh 517507, India; ^3^Department of Pharmacology and Toxicology, University of Kansas, Lawrence, KS 66047, USA; ^4^Department of Biotechnology, Sri Venkateswara Institute of Medical Sciences, Tirupati, Andhra Pradesh 517507, India

## Abstract

Glucokinase (GK) is the predominant hexokinase that acts as glucose sensor and catalyses the formation of Glucose-6-phosphate. The mutations in GK gene influence the affinity for glucose and lead to altered glucose levels in blood causing maturity onset diabetes of the young type 2 (MODY2) condition, which is one of the prominent reasons of type 2 diabetic condition. In view of the importance of mutated GK resulting in hyperglycemic condition, in the present study, molecular dynamics simulations were carried out in intact and 256 E-K mutated GK structures and their energy values and conformational variations were correlated. Energy variations were observed in mutated GK (3500 Kcal/mol) structure with respect to intact GK (5000 Kcal/mol), and it showed increased **γ**-turns, decreased **β**-turns, and more helix-helix interactions that affected substrate binding region where its volume increased from 1089.152 Å^2^ to 1246.353 Å^2^. Molecular docking study revealed variation in docking scores (intact = −12.199 and mutated = −8.383) and binding mode of glucose in the active site of mutated GK where the involvement of A53, S54, K56, K256, D262 and Q286 has resulted in poor glucose binding which probably explains the loss of catalytic activity and the consequent prevailing of high glucose levels in MODY2 condition.

## 1. Introduction

Type 2 diabetic condition is the increase in blood glucose levels and is due to many reasons; one of the most important factor being MODY2 condition, which is characterized at an early age and is an autosomal dominant inherited disorder [1]. Glucokinase (GK) is one of the potential candidate genes for type 2 diabetes acting through elevated fasting plasma glucose. It is a glucose sensing enzyme that catalyses the formation of glucose-6-phosphate from glucose by utilizing one molecule of ATP and that determines the threshold for glucose-stimulated insulin secretion in islets and controls gluconeogenesis and glycogen synthesis in hepatocytes. It can regulate the insulin secretion and integration of hepatic intermediatory metabolism [2]. GK gene is 52.15 kilo bases (kb) in length and is present on Chromosome 7 p13 with 12 exons and produces a transcript of 2.7 kb. A number of reports suggest that the existence of mutations in the coding region of GK is associated with MODY2 [3–11]. The mutated structures show variation in the affinity for binding with glucose, which may affect the kinetics of GK [12, 13]. In order to assess the mutations in GK affecting the catalysis process, *in silico* mutagenic studies will help in revealing the effect of structural and functional variations with respect to mutations in the enzyme such that the same can be exploited to explain the MODY2 condition in type 2 diabetic patients. Molecular dynamics simulation techniques can be applied to study the behavior of both intact and mutated GK structures at any specified conditions, which can be used to investigate its specific molecular interaction in the system [14–16]. The dynamic simulations can explain the interaction and charge distribution of GK using density functional theory calculations in both intact and mutated structures [17]. This technique can also explain the impact of environmental conditions such as solvation and temperature on the GK conformations and energy changes which are of fundamental importance to describe the function and activity. The impact of every mutation on GK conformation can be clearly studied within a very less time. The biochemical function of any protein is defined by its 3D structures, and under physiological conditions, the 3D structures of protein are defined by its component residues among which each residue is having its specific impact on the conformation of the protein. These residues have a primary effect on the rate of protein folding, noncovalent interactions, and kinetic stability. Any mutations in the protein will reflect the variations in the biochemical function of the protein [18]. Determining such key residues would greatly enhance to understand the stability and reactivity of GK under normal and MODY2 condition [19]. Mutations that disrupt overall structure and dynamics can often have drastic functional consequences. The knowledge of structure and function relationship combined with the number of solved structures with no biochemical annotations has motivated the development of computational tools for the prediction of molecular function using sequence and structural information [20]. The identification and analysis of such residues will give an important insight into the structure-function correlations. 

Hence, the present study is aimed to identify the impact of an active site mutation 256 E-K and its influenced regions, which will give a better idea on the activity of both intact and mutated GK. There was a survey by Bell et al. in 1996, indicating the natural occurrence of 256 E-K mutation first time in a population with MODY2 condition, and even they reported the altered activity of GK under mutated condition [21]. Molnes et al. reported in their site-directed mutagenic study that replacement of Glu with Lys/Ala at the 256th position resulted in enzyme forms that did not bind with *α*-D-glucose at a concentration of 200 mM and was essentially catalytically inactive [22]. Gidh-Jain et al. induced this mutation in human *β*-Cell GK by *in vitro* site directed mutagenesis and expressed in *Escherichia coli*, and they observed changes in enzyme activity including a decrease in *V*_max_ and/or increase in *K*_*m*_ for glucose [12]. We analyzed the impact of this active site mutation on the conformational fluctuations of GK and most interestingly into active site variations through molecular dynamics and docking. We observed variations in both the affinity and the binding mode of glucose in the active site along with energy fluctuations that eventually results in the loss of catalytic activity. Our study is strongly supported by the functional analysis done by the previous researchers explained previously.

## 2. Materials and Methods

All the molecular dynamics simulations and molecular docking studies were carried out in molecular operating environment software tool (MOE 2011.10. Chemical Computing Group Inc.).

### 2.1. Preparation of Intact Glucokinase Structure

The X-ray crystallographic structure of GK (PDB ID: 3F9M) at resolution of 1.5 Å was retrieved from Protein Data Bank (http://www.rcsb.org/pdb/home/home.do), which is a huge repository of three-dimensional structures of macromolecules [23]. The water molecules and heteroatoms were removed, polar hydrogens were added, and the structure was protonated. Energy minimization was carried out in MMFF94x force filed at root mean square gradient of 0.05.

### 2.2. Preparation of Mutated Glucokinase Structure

The MODY2 mutation at the 256th position that was reported in GK entry (ID: P35557) of UniProt database [24] and also in previous studies [12, 21, 22] was introduced where Glutamate was replaced with Lysine residue into the energy minimized intact GK structure, and again energy minimization was carried out with the previously explained conditions.

### 2.3. Molecular Dynamics Studies of Energy-Minimized Intact and Mutated GK Structures

The energy minimized conformations of both intact and mutated GK structures were subjected to molecular dynamics simulations individually in the same force field. The NPT (number of particles, pressure, and temperature) statistical ensemble in which the simulations generate stable conformations was specified, and both temperature and pressure were held fixed. The algorithm Nose-Poincare-Anderson (NPA) was specified to solve the equations of motion during simulations. This method is the most the accurate and sensitive, and, it generates true ensemble trajectories. The initial temperature was set to 30 K and increased to a run time temperature of 300 K, and pressure was set to 101 kPa. The heat time was set at 0 picoseconds (ps), the total run time of simulations was carried out for 10 nanoseconds (ns) and the final cool time was set to 0 ps. The constraints were applied on light bonds, and a time step of 0.002 ps was used to discretize the equations of motion. The position, velocity, and acceleration of the trajectories were saved for each 0.5 ps. The energy values of each conformation were plotted as graphs to observe the energy variations among intact and mutated GK.

### 2.4. PDBsum Analysis

PDBsum is a web-based database mainly providing the pictorial summaries of the 3D structures of proteins and their detailed structural analysis [25, 26]. The simulated structures obtained at the end of simulation period were submitted to PDBsum to identify the conformational variations that aroused due to introduction of mutation with respect to intact GK structure. The pictorial representation of mutated structure was correlated with intact structure, and conformational variations were identified.

### 2.5. Structural Alignment

The structural alignment task was carried out by PyMol software tool using align command [27]. The mutated structure was superimposed with intact structure to get a clear insight about the conformational fluctuations, especially in substrate binding regions. The active site residues, that is, T168, K169, N204, D205, N231, E256, and E290, were identified from PDBsum ligand interaction page of GK entry (http://www.ebi.ac.uk/thornton-srv/databases/cgi-bin/pdbsum/GetPage.pl?pdbcode=3f9m&template=ligands.html&l=1.1). The surface volumes of substrate binding cavities were measured to find out the volume differences.

### 2.6. Binding Mode Analysis

A comparative molecular docking analysis was carried out to know the binding mode of glucose in the active site, with both intact and mutated structures using MOE dock tool to obtain a population of possible conformations and orientations for glucose at the binding site. Glucose three-dimensional structure was constructed and optimized in MOE working environment. Initially, the simulated and stabilized trajectory of intact GK structure obtained at the end of the simulations was loaded into MOE. The binding site was defined with the residues T168, K169, N204, D205, N231, E256, and E290, and glucose was specified as ligand. Molecular docking was carried out into the specified binding site using triangle matcher docking placement methodology where the poses are generated by aligning ligand triplets of atoms on triplets of alpha spheres of receptor in a systemic way. A dock database was generated containing 30 docked conformations of the receptor and ligand. Londong dG scoring methodology was applied that estimates the free binding energy of the ligand from a given pose and ranks the docked conformations. The total docked conformations were subjected to refinement in the same force field and rescored using the same scoring function. Duplicates were removed from the final list of docked conformations. After docking process, the conformation with the lowest docking score was chosen for further study and analysis.

The same procedure was also carried out separately for the mutated GK docking process, but among the active site residues specified previously there is Lysine residue at the 256th position, and the remaining residues are same.

### 2.7. Molecular Dynamics Studies of Receptor-Ligand Complexes

The docking complexes of both intact and mutated GK-glucose complexes were subjected to molecular dynamics simulations for 10 ns individually with the same parameters specified previously for GK simulations alone. The energy values of both complexes were plotted as graphs at the end of the simulations to observe the variation. The conformations of ligand and its interaction with active site residues during simulations were analyzed at each 500 ps for both intact and mutated GK-glucose complexes.

## 3. Results

**Figure 1:**
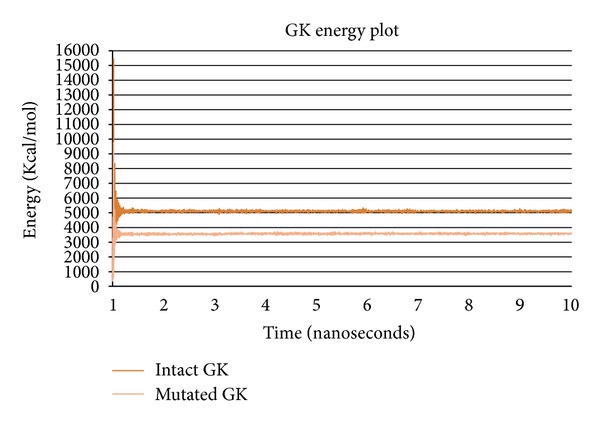
GK energy plot showing the energy transitions of intact and mutated GK structures during molecular dynamics simulations for a period of 10 ns. Intact GK conformation is stabilized around the energy levels of 5000 Kcal/mol and mutated GK around 3500 Kcal/mol.

**Figure 2:**
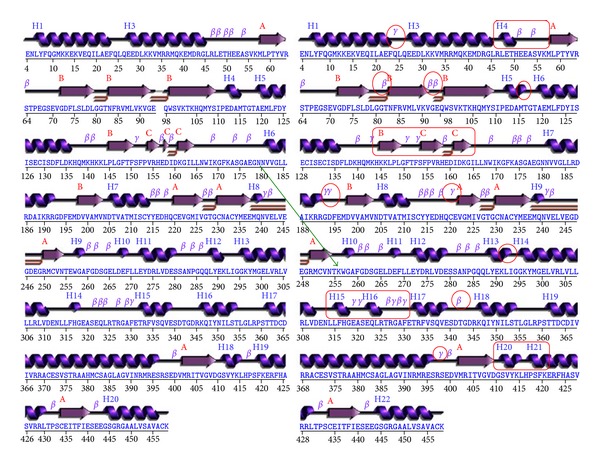
PDBsum analysis of intact GK structure (left) and 256 E-K mutated GK structure (right). The changes in the secondary structure conformations of mutated structure are shown in red-colored circles. These changes are due to mutation at position 256 where Glutamate is replaced with Lysine residue (indicated with green arrow).

The stabilized trajectories of intact and mutated GK structures obtained at the end of simulations were observed for their energy variations. The intact GK structure with an initial energy of 525.966 Kcal/mol was stabilized around 5000 Kcal/mol while, the mutated GK structure with an initial energy of 365.061 Kcal/mol was stabilized around 3500 Kcal/mol in a 10 ns of simulation (Figure 1). This energy variation is the result of the substitution of E with K at the 256th position, and this mutation showed its effect not only on the energy of the GK but also on the secondary structure conformation. The mutated GK structure showed increased *γ* turns, decreased *β* turns and more helix-helix interactions compared to intact GK structure as revealed from PDBsum analysis, indicating that 256 E-K, that is, acidic to basic amino acid replacement has profound effect on the GK conformation (Figure 2, Table 1).

**Figure 3:**
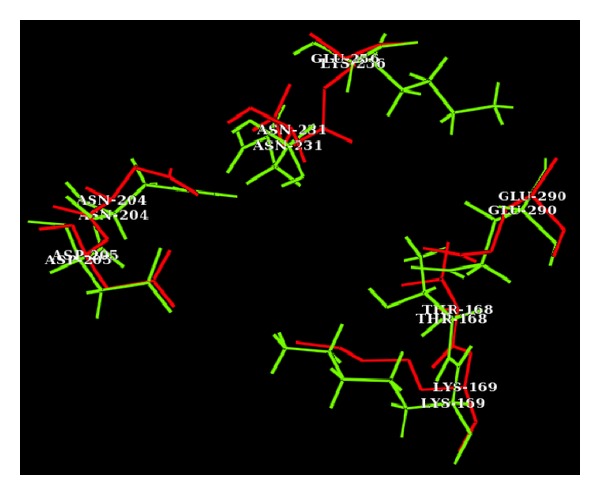
Superimposition of substrate binding regions of intact (red) and 256 E-K mutated (green) GK structures. The distance between the superimposed residues explains the variation in volume and surface area of substrate binding region, which in turn influences the binding affinity with glucose.

The superimposition of substrate binding site of mutated GK with intact GK showed distinct changes which is correlated with their molecular surface area. The intact GK substrate binding site showed a surface area of 1089.152 Å^2^ where glucose binds and fits into the cavity, and it was changed to 1246.353 Å^2^ in the mutated structure (Figure 3). 

**Figure 4:**
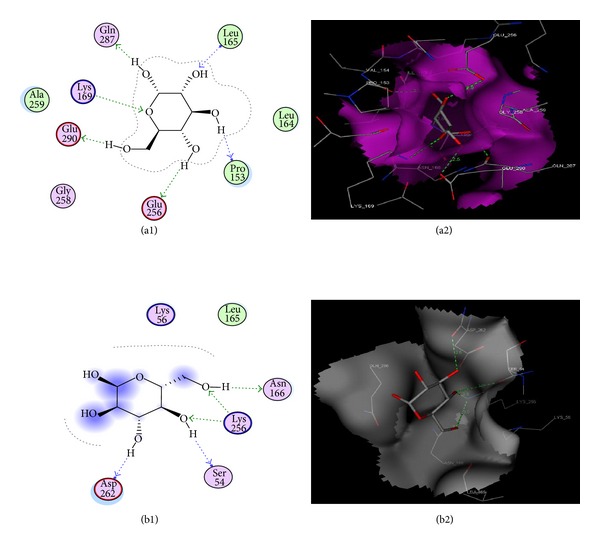
Binding mode of glucose with intact and mutated GK active sites after molecular docking. (a1) Two-dimensional linear representation of the glucose interaction with intact GK active site residues showing 6 hydrogen bonds. (a2) Three-dimensional graphical representation of glucose interaction found to be sit in the active site cavity with hydrogen bond interactions. (b1) Two-dimensional linear representation of glucose interaction with mutated GK active site residues showing 5 hydrogen bonds. The blue-colored shade represents the solvent exposure area of glucose molecule. (b2) Three-dimensional graphical representation of glucose interaction found to be on the surface of active site cavity with limited hydrogen bond interactions.

Further, molecular docking analysis revealed that glucose is binding with the intact GK active site forming hydrogen bonds with P153, L165, K169, E256, Q287, and E290 residues while in mutated GK showed hydrogen bonds with S54, N166, K256 and D262 residues. The docking scores −12.199 and −8.383 of intact GK and mutated GK, respectively, showed that the affinity of binding of glucose decreased in mutated GK (Figure 4). Here, the mutated residue lysine at position 256 is found to be interacting with glucose molecule forming two hydrogen bonds. There is a drastic variation in the binding mode of glucose with intact GK active site where it was found to be sitting in the cavity and showed no interaction with the solvent, whereas in the mutated GK active site, the glucose molecule was found to be on the surface of the cavity and was interacting with the solvent. These variations in the glucose interaction were due to the mutation generated in the GK molecule (Table 2). 

**Figure 5:**
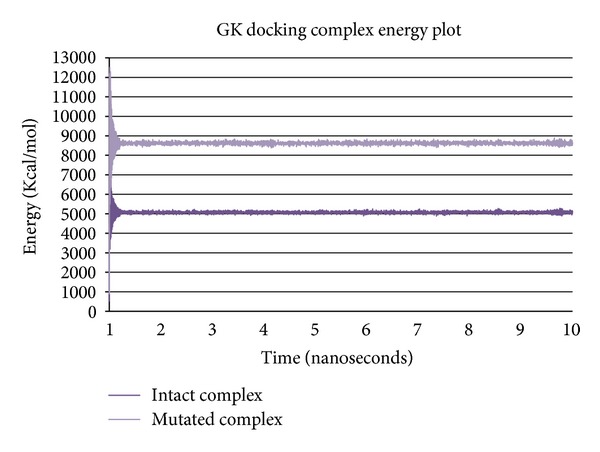
Energy transition plot of intact and mutated GK docking complexes during molecular dynamics simulations for a period of 10 ns. Intact GK docking complex is stabilized around the energy levels of 5000 Kcal/mol and mutated GK docking complex around 8800 Kcal/mol.

The comparative molecular dynamics simulations results of the docking complexes of both intact and mutated GK showed variations in energy transitions and conformations during simulation period. The intact GK docking complex showed stability around energy levels of 5000 Kcal/mol which is equal to the energy transitions of intact GK simulations, and no energy fluctuations were observed even after docking process, while mutated GK docking complex showed variations in energy levels of 8600 Kcal/mol; however, the mutated GK alone showed energy levels around 3500 Kcal/mol (Figure 5). These results clearly indicated that energy levels were the same in intact GK when it is docked with glucose, while extensive variation in energy levels with mutated GK is due to the change in the acidic to basic amino acid which probably prevented the release of H^+^ ions in the phosphorylation reaction. Further, the conformational analysis at every 500 ps for both intact and mutated complexes, the binding orientations of glucose, and its interaction with the specific active site residues at specific time period of simulations explain the binding affinity variations of glucose to the active site (Table 3) (see Supplementary information in Tables S1 and S2 in the Supplementary Material available online at http://dx.doi.org/10.1155/2013/264793).

Majority of the conformations of intact GK complex showed the major contribution by K169 to bind with glucose followed by L165, N166, and Q256. A very less frequency of interaction was observed with P153, Q287, and E290. Mutated GK docking complex conformations revealed that only N166 and Q287 were found to be interacting commonly as the intact GK. The new residues such as A53, S54, K56, K256, D262, and Q286 that are in the surrounding area of the active site came into interaction with glucose among which the major contribution was made by D262 followed by Q286, and a very less frequency of interaction was made by S54, K56, and K256 residues. Interaction of glucose with these residues in mutated GK making it come out from the binding site cavity and showing interaction with solvent. This may be a responsible factor along with drastic energy variations bringing instability in GK-glucose complex which may result in poor binding of glucose and may also result in the disassociation of the complex. Such a mutation is observed in MODY2 condition, which, therefore, explains the loss of catalytic activity resulting in high glucose condition in type 2 diabetes.

## 4. Discussion

Natural mutations in GK gene result in poor affinity towards glucose resulting in high blood glucose levels, which is one of the condition in type 2 diabetes and these mutations are explained as MODY2 mutations. Basically, the mutations are observed throughout the gene so far. Increased type 2 diabetic population all over the world with different MODY2 mutations in GK gene showing altered affinity towards glucose could be fatal in such patients. In order to elucidate the probable occurrence of such mutations and their impact on GK catalysis, in the present study, we concentrated on an active site MODY2 mutation 256 E-K and carried out comparative molecular dynamics simulations and molecular docking studies. For this purpose, the intact and mutated GK structures were simulated and submitted to PDBsum for the conformational analysis and observed extensive conformational variations not only in the active site but also throughout the mutated GK structure. The active site variations were correlated with its molecular surface area, which in turn explains decreased glucose binding in the mutated structure. This variation of glucose binding affects the catalytic properties of GK. This mutation is not only affecting the conformation of the structure but also results in extremely variable energy levels.

Thus, this kind of variations in both energies and conformations clearly explains not only the decreased affinity for glucose but also increased blood glucose levels in the patients affected with MODY2 mutation. Zhang et al. also demonstrated this kind of study where they explained the importance of K169 residue in the GK catalytic mechanism with the help of molecular dynamics simulations, and they even verified their prediction by experimental mutagenesis and enzymatic analysis to provide a strong evidence for the pathogenic mechanism of MODY2 condition [16]. In the same way, this study can provide the evidence for altered catalytic mechanism of each MODY2 mutated GK. Ramirez et al. also studied in the same manner to identify the mutation inducing variations in the active site of Haemoglobin I from *Lucina pectinata*, and they analyzed the ligand binding kinetics that plays major role in the stabilization process of binding site [28].

Figure 2 can explain clear comparative pictorial variations in the mutated GK secondary structural conformation where two new *α* helices were formed, three *β* turns were lost, and ten new *γ* turns were generated. To observe the impact of this mutation on the substrate binding site, the simulated structures of intact and mutated GK were superimposed, and the change in the cavity volume was clearly observed providing the reason for positional fluctuations of glucose. Figure 4 shows the interaction of glucose with the substrate binding sites of intact and mutated GK structures where the positional changes are clearly observed. This was strengthened by molecular docking analysis where we observed the variation in docking scores and binding mode of glucose among intact and mutated GK structures. Comparatively, the lowest docking score was observed with intact GK which explains the stronger affinity of glucose to the active site than in mutated one. 

The molecular dynamics simulations of intact and mutated GK-glucose docking complexes revealed the energy transition variations where the intact GK showed no significant variation even after docking, but mutated GK showed higher energy levels after docking process. Such higher energy levels result in less affinity between enzyme and substrate and may also cause the dissociation of complex, thereby the rate of reaction will be reduced. The intact and mutated docking conformations are showing three common interacting residues, that is, L165, N166, and Q287 (Table 3) indicating the importance of these residues in the substrate binding mechanism and in the positional shift of glucose molecule. The remaining residues P153, K169, E256, and E290 that were found to be interacting with glucose in the intact GK active site lost their interaction because of conformational variations due to mutation in the active site where the other new residues A53, S54, K56, K256, D262, and Q286 came into interaction. Because of this, there is drastic variation in the conformation of active site resulting in poor binding of glucose and which eventually resulted in loss of catalytic activity. The significance of K169 residue in the catalytic activity of GK was already experimentally proved [16], so loss of interaction of such key residues of catalysis in the mutated GK could affect the catalytic mechanism of glucose phosphorylation in the active site. This may be explained with the variation seen in docking scores where the mutated GK showed higher docking score than the intact GK that cleared the reduced affinity for glucose. 

These variations in mutated structure probably affect the binding affinity of glucose and catalytic activity of GK that will finally affect the phosphorylation and utilization of glucose and in turn results in the hyperglycemic condition. Such variations are characteristic features observed in MODY2. Thus, this study clearly explains the reasons for the increased blood glucose levels due to altered catalytic activities of GK in MODY2 condition.

## 5. Conclusion

The conformational fluctuations that aroused in the structure of GK are due to the mutation, which may alter its affinity for binding with glucose. This study had best explained the conformational variations of mutated GK structure, in both functional and nonfunctional regions. Finally, it provided a strong reason for the affinity changes in terms of both energy and docking score. Further, the 256 E-K mutation has profound effect on the conformational variation of active site resulting in poor binding of glucose and loss of catalytic activity.

## Supplementary Material

Table S1: The simulation time period of each trajectory is shown in the corner of each cell of the table. The dotted arrows are the hydrogen bond interactions between glucose and intact GK active site residues.
Table S2: The simulation time period of each trajectory is shown in the corner of each cell of the table. The dotted arrows are the hydrogen bond interactions between glucose and mutated GK active site residues. The blue shaded region around the glucose molecule indicates its solvent contacts which are not observed in the intact GK-glucose docking conformations.

## Figures and Tables

**Table 1:** PDBsum analysis showing the variations in secondary structural conformations of intact and mutated GK structures.

Secondary conformation^a^	Intact GK^b^	Mutated GK^c^
Sheets	3	3
Beta alpha beta unit	1	1
Beta hairpins	5	5
Beta hairpins	5	4
Strands	13	13
Helices	20	22
Helix-helix interactions	24	40
*β* turns	34	31
*γ* turn	3	13

^a^Type of secondary conformation.

^b^Number of respective secondary conformations observed in intact GK.

^c^Number of respective secondary conformations observed in mutated GK.

**Table 2:** Molecular docking of glucose into the active site cavity of intact and mutated GK. Docking score shown in the second column indicates the binding affinity of glucose to the active site. The lower is the score, the higher will be the stability of the complex. The interacting active site residues of GK that are involved in formation of hydrogen bonds with glucose are shown in the fourth column, and the respective hydrogen bond lengths are indicated in Angstroms in the last column.

GK structure	Docking score	No. H-bonds	Interacting residue of GK	H-bond length (Å)
			P 153	1.49
			E 256	2.04
Intact	−12.199	6	Q 287	1.45
			E 290	1.58
			L 165	2.63
			K 169	2.95

			S 54	2.45
			N 166	1.54
Mutated	−8.383	5	D 262	1.69
			K 256	2.46
			K 256	3.00

**Table 3:** Interaction of glucose with active site of intact and mutated GK and energy transitions of GK-glucose complexes during molecular dynamics simulations for a period of 10 ns.

Simulation^a^ period (ps)	No. H-bonds^b^		Interacting residues of GK active site^c^		Energy of the complex^d^ (Kcal/mol)	
	Intact	Mutated	Intact	Mutated	Intact	Mutated
0	6	5	**P153**, *L165*, **K169**, **E256**, **E290**, *Q287 *	***S54, D262, *** *N166*, ***K256, K256 ***	527.75	366.85
500	5	8	**P153**, *L165*, *N166*, **K169**, *Q287 *	***A53, K56, *** *N166*, ***Q286, Q286, D262, D262, D262 ***	5135.83	8536.07
1000	5	6	**P153**, *L165*, *N166*, **K169**, *Q287 *	***A53, *** *N166*, ***K256, D262, D262, Q286 ***	5054.68	8600.38
1500	6	8	**P153**, *L165*, *N166*, **K169**, *Q287*, **E290**	***A53, *** *L165*, ***K256, K256, D262, D262, D262, Q286 ***	5066.78	8553.16
2000	6	4	*L165*, *N166*, **K169**, **E256**, *Q287*, *Q287 *	***A53, D262, D262, Q286 ***	5112.49	8625.42
2500	8	4	*L165*, *L165*, *N166*, *N166*, **K169**, **K169**, **E256**, *Q287 *	***A53, K256, D262, D262 ***	5094.22	8536.71
3000	6	4	*L165*, *N166*, **K169**, **K169**, **E256**, **E290**	***A53, D262, D262, Q286 ***	5087.11	8534.96
3500	8	6	*L165*, *L165*, *N166*, *N166*, **K169**, **K169**, **K169**, **E256**	***A53, S54, D262, D262, D262, Q286 ***	5154.06	8633.15
4000	8	7	*L165*, *L165*, *N166*, *N166*, **K169**, **K169**, **K169**, **E256**	***A53, D262, D262, D262, Q286, Q286, *** *Q287 *	5088.23	8608.42
4500	8	7	*L165*, *N166*, *N166*, **K169**, **K169**, **K169**, **E256**, *Q287 *	***A53, S54, D262, D262, D262, Q286, *** *Q287 *	5014.87	8579.74
5000	8	5	*L165*, *L165*, *N166*, *N166*, **K169**, **K169**, **K169**, **E256**	***A53, D262, D262, Q286, Q286 ***	5078.62	8521.10
5500	6	5	*L165*, *N166*, **K169**, **K169**, **K169**, **E256**	***A53, D262, D262, D262, Q286 ***	5087.78	8548.05
6000	8	5	*L165*, *L165*, *N166*, *N166*, **K169**, **K169**, **K169**, **E256**	***A53, D262, D262, D262, Q286 ***	5116.11	8566.65
6500	8	7	*L165*, *L165*, *N166*, *N166*, **K169**, **K169**, **K169**, **E256**	***A53, S54, D262, D262, D262, Q286, Q286 ***	5059.85	8542.55
7000	7	6	*L165*, *N166*, *N166*, **K169**, **K169**, **K169**, **E256**	***A53, D262, D262, D262, Q286, Q286 ***	5030.70	8446.74
7500	8	6	*L165*, *L165*, *N166*, *N166*, **K169**, **K169**, **K169**, **E256**	***A53, S54, D262, D262, D262, Q286 ***	5063.48	8597.48
8000	4	7	*N166*, *N166*, **K169**, **E256**	***A53, S54, D262, D262, D262, Q286, *** *Q287 *	5106.54	8658.68
8500	8	5	*L165*, *L165*, *N166*, *N166*, **K169**, **K169**, **E256**, *Q287 *	***A53, D262, D262, D262, Q286 ***	5104.12	8548.79
9000	6	7	*L165*, *N166*, **K169**, **K169**, **K169**, **E256**	***A53, S54, D262, D262, D262, Q286, *** *Q287 *	5127.97	8518.22
9500	7	4	*L165*, *L165*, *N166*, **K169**, **K169**, **K169**, **E256**	***A53, D262, D262, Q286 ***	5105.28	8574.21
10000	8	6	*L165*, *L165*, *N166*, *N166*, **K169**, **K169**, **K169**, **E256**	***A53, S54, D262, D262, D262, Q286 ***	5160.90	8522.43

^a^Duration of simulation period where the respective conformation was analyzed.

^b^Number of hydrogen bonds formed between the glucose and active site residues of intact and mutated GK.

^c^Interacting residues of intact and mutated GK during simulations in a specified conformation. The residues in bold are active site residues that are interacting with glucose specifically from intact GK, the residues in italic are found to be interacting with glucose in both intact and mutated GK, and the residues in bold italic are found to be interacting with glucose in mutated GK only.

^d^Energies of the docking complexes of intact and mutated GK at specified simulation periods.

## References

[1] Hattersley AT, Turner RC, Permutt MA (1992). Linkage of type 2 diabetes to the glucokinase gene. *The Lancet*.

[2] Agius L (2009). Targeting hepatic glucokinase in type 2 diabetes: weighing the benefits and risks. *Diabetes*.

[3] Stoffel M, Froguel P, Takeda J (1992). Human glucokinase gene: Isolation, characterization, and identification of two missense mutations linked to early-onset non-insulin-dependent (type 2) diabetes mellitus. *Proceedings of the National Academy of Sciences of the United States of America*.

[4] Stoffel M, Patel P, Lo YMD (1992). Missense glucokinase mutation in maturity-onset diabetes of the young and mutation screening in late-onset diabetes. *Nature Genetics*.

[5] Sakura H, Eto K, Kadowaki H (1992). Structure of the human glucokinase gene and identification of a missense mutation in a Japanese patient with early-onset non-insulin-dependent diabetes mellitus. *Journal of Clinical Endocrinology and Metabolism*.

[6] Hager J, Blanche H, Sun F (1994). Six mutations in the glucokinase gene identified in MODY by using a nonradioactive sensitive screening technique. *Diabetes*.

[7] Guazzini B, Gaffi D, Mainieri D (1998). Three novel missense mutations in the glucokinase gene (G80S; E221K; G227C) in Italian subjects with maturity-onset diabetes of the young (MODY). Mutations in brief no. 162. Online. *Human Mutation*.

[8] Hattersley AT, Beards F, Ballantyne E, Appleton M, Harvey R, Ellard S (1998). Mutations in the glucokinase gene of the fetus result in reduced birth weight. *Nature Genetics*.

[9] Ng MCY, Cockburn BN, Lindner TH (1999). Molecular genetics of diabetes mellitus in chinese subjects: Identification of mutations in glucokinase and hepatocyte nuclear factor-1*α* genes in patients with early-onset type 2 diabetes mellitus/MODY. *Diabetic Medicine*.

[10] Nam JH, Lee HC, Kim YH (2000). Identification of glucokinase mutation in subjects with post-renal transplantation diabetes mellitus. *Diabetes Research and Clinical Practice*.

[11] Njølstad PR, Søvik O, Cuesta-Muñoz A (2001). Neonatal diabetes mellitus due to complete glucokinase deficiency. *The New England Journal of Medicine*.

[12] Gidh-Jain M, Takeda J, Xu LZ (1993). Glucokinase mutations associated with non-insulin-dependent (type 2) diabetes mellitus have decreased enzymatic activity: implications for structure/function relationships. *Proceedings of the National Academy of Sciences of the United States of America*.

[13] Stoffel M, Bell KL, Blackburn CL (1993). Identification of glucokinase mutations in subjects with gestational diabetes mellitus. *Diabetes*.

[14] Merino F, Guixé V (2008). Specificity evolution of the ADP-dependent sugar kinase family—in silico studies of the glucokinase/phosphofructokinase bifunctional enzyme from *Methanocaldococcus jannaschii*. *FEBS Journal*.

[15] de Oliveira CAF, Zissen M, Mongon J, Mccammon JA (2007). Molecular dynamics simulations of metalloproteinases types 2 and 3 reveal differences in the dynamic behavior of the S1′ binding pocket. *Current Pharmaceutical Design*.

[16] Zhang J, Li C, Shi T, Chen K, Shen X, Jiang H (2009). Lys169 of human glucokinase is a determinant for glucose phosphorylation: implication for the atomic mechanism of glucokinase catalysis. *PLoS ONE*.

[17] Nagarajan S, Rajadas J, Malar EJP (2010). Density functional theory analysis and spectral studies on amyloid peptide A*β*(28-35) and its mutants A30G and A30I. *Journal of Structural Biology*.

[18] Takeda J, Gidh-Jain M, Xu LZ (1993). Structure/function studies of human *β*-cell glucokinase. Enzymatic properties of a sequence polymorphism, mutations associated with diabetes, and other site-directed mutants. *The Journal of Biological Chemistry*.

[19] Dosztányi Z, Magyar C, Tusnády GE, Cserzo M, Fiser A, Simon I (2003). Servers for sequence-structure relationship analysis and prediction. *Nucleic Acids Research*.

[20] Sadowski MI, Jones DT (2009). The sequence-structure relationship and protein function prediction. *Current Opinion in Structural Biology*.

[21] Bell GI, Pilkis SJ, Weber IT, Polonsky KS (1996). Glucokinase mutations, insulin secretion, and diabetes mellitus. *Annual Review of Physiology*.

[22] Molnes J, Bjørkhaug L, Søvik O, Njølstad PR, Flatmark T (2008). Catalytic activation of human glucokinase by substrate binding—residue contacts involved in the binding of D-glucose to the super-open form and conformational transitions. *FEBS Journal*.

[23] Berman HM, Westbrook J, Feng Z (2000). The protein data bank. *Nucleic Acids Research*.

[24] Wu CH, Apweiler R, Bairoch A (2006). The universal protein resource (UniProt): an expanding universe of protein information. *Nucleic Acids Research*.

[25] Laskowski RA (2001). PDBsum: summaries and analyses of PDB structures. *Nucleic Acids Research*.

[26] Laskowski RA, Hutchinson EG, Michie AD, Wallace AC, Jones ML, Thornton JM (1997). PDBsum: a web-based database of summaries and analyses of all PDB structures. *Trends in Biochemical Sciences*.

[27] Seeliger D, de Groot BL (2010). Ligand docking and binding site analysis with PyMOL and Autodock/Vina. *Journal of Computer-Aided Molecular Design*.

[28] Ramirez E, Cruz A, Rodriguez D (2008). Effects of active site mutations in haemoglobin i from Lucina pectinata: a molecular dynamic study. *Molecular Simulation*.

