# Supplementary material for: Structural Variations of Human Glucokinase Glu256Lys in MODY2 Condition Using Molecular Dynamics Study
Source: Biotechnol Res Int. 2013 Feb 13;2013:264793. doi: 10.1155/2013/264793 (PMC3586473; doi:10.1155/2013/264793)

Supplementary Information

TABLE S1: The conformations and binding orientations of glucose in the active site of intact GK during molecular dynamics simulations. The conformations were analyzed for every 500 ps for a total period of 10 ns.

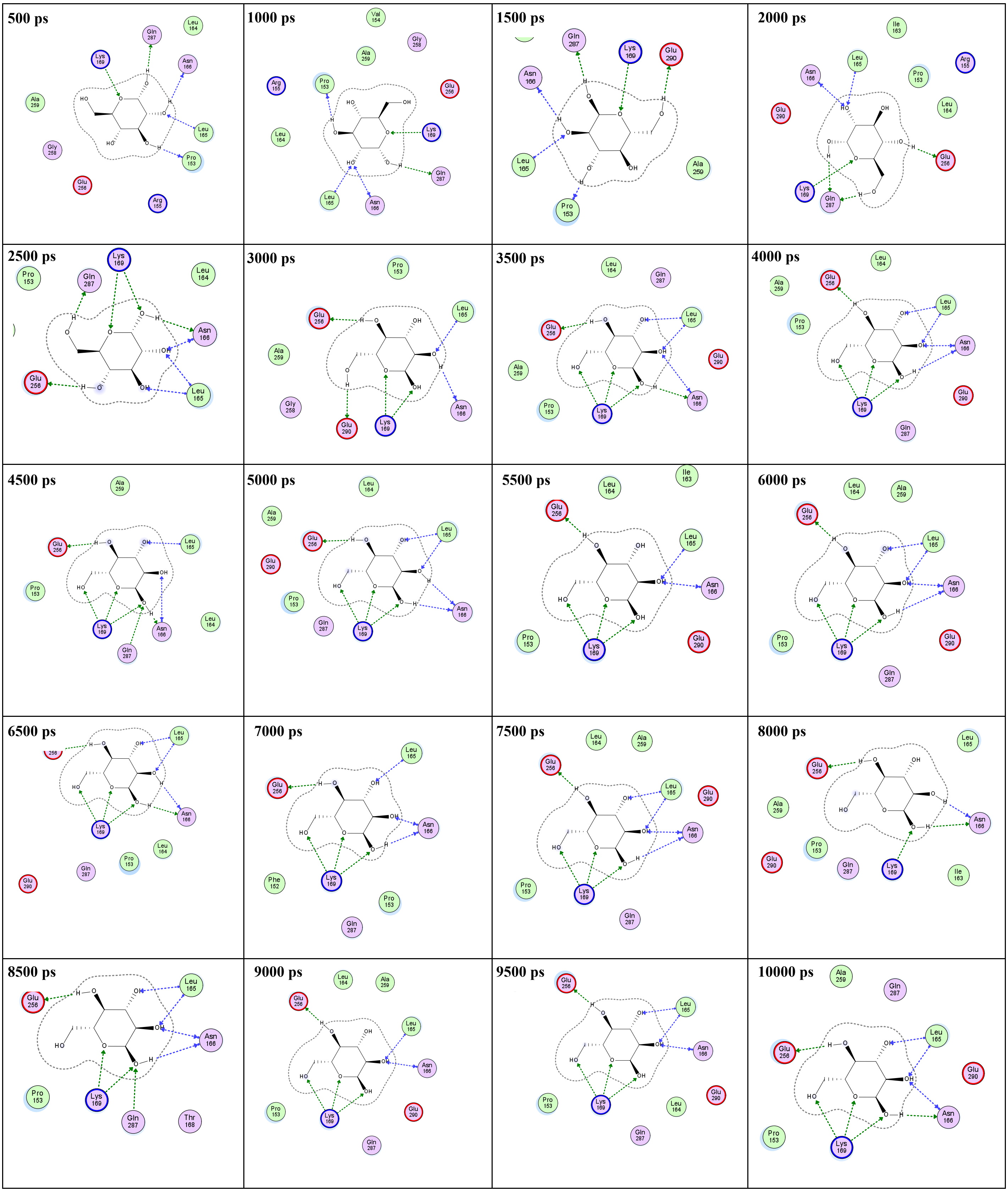

Table S2: The conformations and binding orientations of glucose in the active site of mutated GK during molecular dynamics simulations. The conformations were analyzed for every 500 ps for a total period of 10 ns.

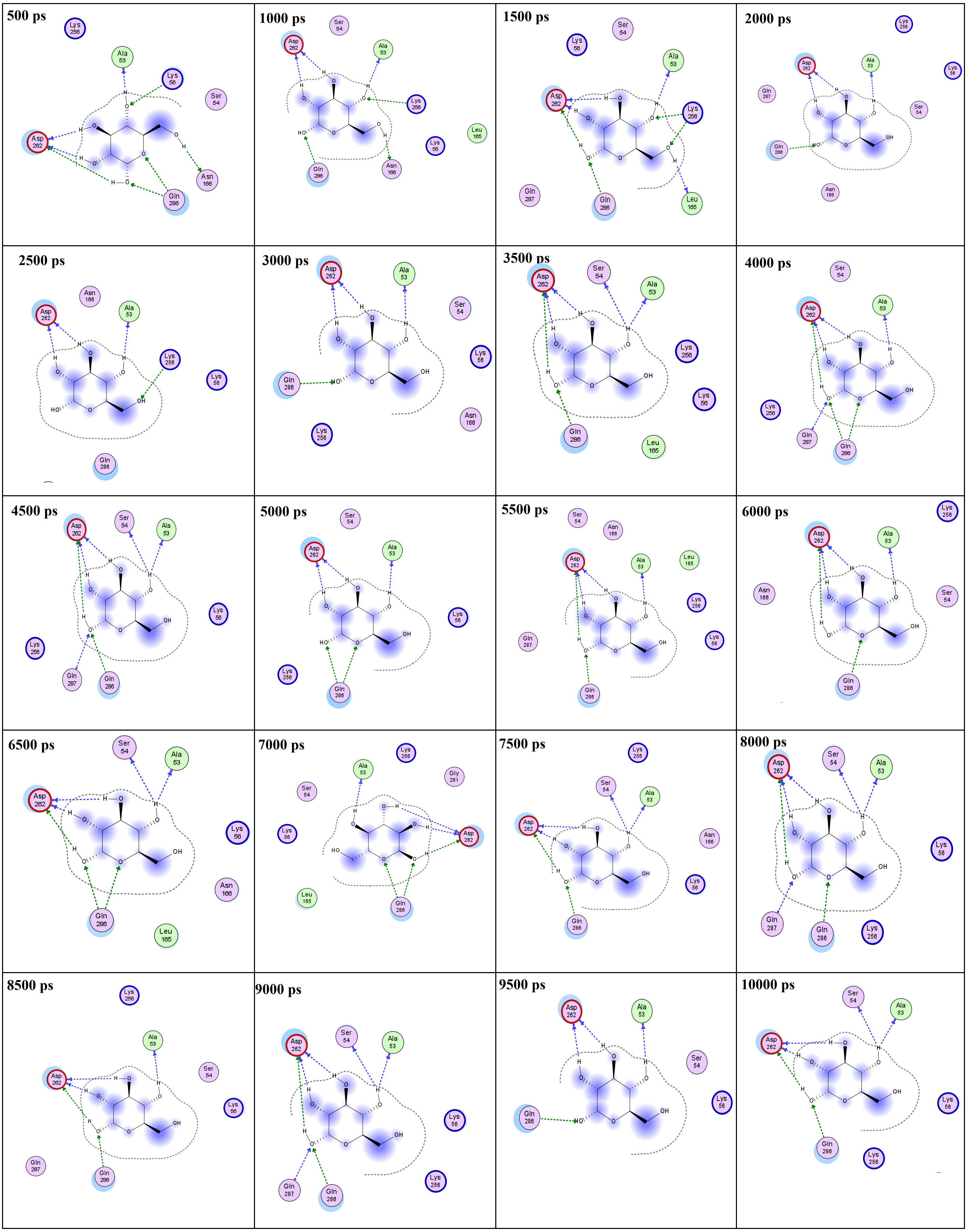

Supplement: Supplementary file 1 — Table S1: The simulation time period of each trajectory is shown in the corner of each cell of the table. The dotted arrows are the hydrogen bond interactions between glucose and intact GK active site residues. Table S2: The simulation time period of each trajectory is shown in the corner of each cell of the table. The dotted arrows are the hydrogen bond interactions between glucose and mutated GK active site residues. The blue shaded region around the glucose molecule indicates its solvent contacts which are not observed in the intact GK-glucose docking conformations. [file 264793.f1.pdf]
